# Influence of Particle Size and Drug Load on Amorphous Solid Dispersions Containing pH-Dependent Soluble Polymers and the Weak Base Ketoconazole

**DOI:** 10.1208/s12249-020-01914-7

**Published:** 2021-01-12

**Authors:** Marius Monschke, Kevin Kayser, Karl G. Wagner

**Affiliations:** Department of Pharmaceutical Technology and Biopharmaceutics, University of Bonn, Gerhard-Domagk-Str. 3, 53121 Bonn, Germany

**Keywords:** Amorphous solid dispersions, Hot-melt extrusion, pH-dependent soluble polymers, Supersaturation

## Abstract

Among the great number of poorly soluble drugs in pharmaceutical development, most of them are weak bases. Typically, they readily dissolve in an acidic environment but are prone to precipitation at elevated pH. This was aimed to be counteracted by the preparation of amorphous solid dispersions (ASDs) using the pH-dependent soluble polymers methacrylic acid ethylacrylate copolymer (Eudragit L100–55) and hydroxypropylmethylcellulose acetate succinate (HPMCAS) *via* hot-melt extrusion. The hot-melt extruded ASDs were of amorphous nature and single phased with the presence of specific interactions between drug and polymer as revealed by X-ray powder diffraction (XRPD), differential scanning calorimetry (DSC), and Fourier-transform infrared spectroscopy (FT-IR). The ASDs were milled and classified into six particle size fractions. We investigated the influence of particle size, drug load, and polymer type on the dissolution performance. The best dissolution performance was achieved for the ASD made from Eudragit L100–55 at a drug load of 10%, whereby the dissolution rate was inversely proportional to the particle size. Within a pH-shift dissolution experiment (from pH 1 to pH 6.8), amorphous-amorphous phase separation occurred as a result of exposure to acidic medium which caused markedly reduced dissolution rates at subsequent higher pH values. Phase separation could be prevented by using enteric capsules (Vcaps Enteric®), which provided optimal dissolution profiles for the Eudragit L100–55 ASD at a drug load of 10%.

## Introduction

A fundamental problem in pharmaceutical development is the poor aqueous solubility of most of the new chemical entities. In many cases, the poor solubility entails a low oral bioavailability (1–4). Consequently, there is a need for formulation strategies which are capable to overcome this limitation. In the past, several approaches have been tested to address this by increasing the solubility or the dissolution rate of these compounds (5). Among these formulation approaches, amorphous solid dispersions (ASDs) were successfully applied. ASDs were shown to be able to enhance the oral bioavailability and there are several approved ASD formulations on the marked (6–8). An ASD consists of a drug, which is incorporated into a polymeric matrix, preferably in a molecularly dispersed state (single phase ASDs or solid solution) (9,10). From a physical stability perspective, the polymer could prevent crystallization of the drug. Thereby the drug can be thermodynamically stabilized within the polymer, if the drug is dissolved in the matrix below its solubility limit (11). Alternatively, the drug can also be kinetically stabilized due to decreased molecular mobility (12,13). ASDs usually cause supersaturated solutions upon dissolution as the drug is released out of its molecularly dispersed state facilitated by the high aqueous solubility and good wettability of the hydrophilic polymer (14). Once the drug is brought in solution, the supersaturated solution could also be stabilized by the precipitation inhibitory properties of the polymer through specific and unspecific interactions (15–17). Ultimately, the generation of a supersaturation for a sufficient duration leads to an increased bioavailability (18). Several polymers have been tested as carriers for ASDs, the most commonly used polymers are those with immediate release characteristics, e.g., polyvinylpyrrolidone, copovidone, or hydroxypropyl cellulose (19). In addition, polymers with sustained release properties (20–22) or a pH-dependent solubility have also been applied successfully (23,24). ASDs formulated using pH-dependent soluble polymers as matrix featured several merits. These types of ASDs provided protection of the drug from the gastric juice, or were shown to be independent of the gastric pH value, since the dissolution is supposed to start in the intestinal region (25). Miller et al. (2008) showed that an ASD comprising of a pH-dependent soluble polymer caused a higher oral bioavailability as compared with an ASD based on an immediate release polymer (26).

The most common preparation techniques for ASDs are hot-melt extrusion (melt based) and spray-drying (solvent based) (7). For a hot-melt extrusion process, the material needs to be thermally stable and its melt viscosity should be in an acceptable range, additionally drug and polymer should be miscible in the ratio which is used (27,28). If the requirements for the materials to be extruded are met, one could benefit from the variety of options with regard to the downstream processing. Different shapes of the extrusion strand can be generated by using different types of dies. In particular, the particle size of the ASD can be easily adjusted, by using sufficient milling techniques. Particle sizes are important for the control of the release properties (8). Zhang et al. (2018) compared very fine ASD particles obtained by spray-drying with coarse ASD particles obtained by hot-melt extrusion and they found that the production technique profoundly impacted the dissolution properties (29). Zheng et al. (2019) showed that the dissolution rate were inversely proportional to the particle size (30). However, also the recrystallization kinetics were dependent on the particle size since the extent of supersaturation and the supersaturation generation rate are determinative for the nucleation rate (31,32). These findings indicate a complex relation between ASD particle size and its actual performance. The complexity should even increase if a weakly basic drug is formulated as ASD due to its pH-dependent solubility profile. Weakly basic drugs typically dissolve at low pH and potentially precipitate at elevated pH (above the pK_A_) if the unionized form of the base is poorly soluble. As a consequence, those drugs often exhibit a low oral bioavailability as the intestinal pH is known to be neutral to basic (33–35). Several approaches aimed to overcome that issue by using acidifiers to modulate the microenvironmental pH (36–38). Zecevic et al. (2014) revealed that the enabling effect of an ASD comprising of a poorly soluble weak base together with an immediate release polymer was leveled as the drug precipitated upon transition to regions with elevated pH (24). Therefore, it would be desirable to create a formulation approach from which weakly basic drugs benefit at the intestinal pH region.

In this study, we formulated ASDs containing the poorly soluble weak base ketoconazole (KTZ) using two different pH-dependent soluble polymers, namely, hydroxypropylmethylcellulose acetate succinate (HPMCAS LG) and methacrylic acid ethyl acrylate copolymer (Eudragit L100–55). By using pH-dependent soluble polymers, it was desired to convert the dissolution behavior (low dissolution at low pH and high dissolution at neutral pH) of the weak base KTZ, with the highest possible dissolution performance at neutral pH. However, at gastric pH, the dissolution of a weak base within a pulverized formulation would be high, regardless of the type of the matrix functionality. We hypothesized that the particle size and drug load will be a decisive factor for the dissolution pattern of a weakly basic drug formulated as pH-dependent soluble ASD. Therefore, a variety of particle sizes and two drug loads, respectively, were assessed to find a potential optimum regarding the dissolution performance. There is only limited information available about the influence of polymer type, drug load and particle size on the dissolution behavior of a pH-dependent soluble ASD containing a weakly basic drug. The hot-melt extruded ASDs were analyzed regarding solid state using differential scanning calorimetry (DSC), X-ray powder diffraction (XRPD), and Fourier-transform infrared spectroscopy (FT-IR). Subsequently, the dissolution performance of the ASDs was tested at static pH (6.8). Additionally, a pH-shift dissolution experiment (from acidic to neutral) was performed to study the influence of a biorelevant pH transition.

## Material and Methods

### Materials

The model drug ketoconazole was purchased from Fagron (Glinde, Germany). HPMCAS LG (glass transition temperature: 120°C) was gifted from Shin-Etsu (Tokyo, Japan), and Eudragit L100–55 (glass transition temperature: 120°C) was donated from Evonik (Darmstadt, Germany). PEG 3000 was obtained from Merck (Darmstadt, Germany). Vcaps Enteric® (consisting of HPMCAS) were donated by Capsugel (Morristown, NJ, USA).

### Hot-Melt Extrusion (HME)

Prior extrusion, physical mixtures were blended for 10 min by aid of a turbula mixer (Willy A. Bachofen AG Maschinenfabrik, Switzerland). The ASDs were prepared using a ZE 12 Three-Tec 12 mm co-rotating twin screw extruder (Seon, Switzerland) with five heating zones and a functional length of 25:1 L/D equipped with a 2-mm die. The extruded compositions and the temperature profiles are listed in Table I. The feed rate and screw speed were kept constant at 2 g/min and 100 rpm, respectively. For the formulations containing Eudragit L100–55, 10% PEG 3000 as plasticizer was added to obtain an extrudable blend. This was necessary since Eudragit L100–55 exhibits a very high melt viscosity and preliminary experiments showed that the polymer alone could not be extruded as it caused a motor overload of the extruder.

**Table I:** Compositions of the formulations and extrusion temperature profiles

Composition	Ratio	Temp. [°C]
HPMCAS LG:KTZ	90:10	100/140/140/140/140
	75:25	100/140/140/140/140
L100–55:PEG:KTZ	80:10:10	100/140/140/140/140
	65:10:25	100/140/140/140/140

### Preparation of Different Particle Sizes

In order to investigate the influence of different particle sizes, six different particle fractions were prepared (Table II). For the manufacture of the finer particle fractions (125–45 μm, 250–125 μm and 500–250 μm) a mixer mill 400 (Retsch, Haan, Germany) was used at 30 Hz for 30 s and for the manufacture of the coarser fractions a laboratory hammer mill with an inserted 2 mm sieve was used. Subsequently, the milled materials were classified using a sieve shaker (AS 200, Retsch, Haan, Germany) at an amplitude of 50% for 2 min with six different sieves with mesh sizes according to Table II.

**Table II:** Sieve fractions that were used to classify the milled ASDs

Sieve fraction [μm]	
1500–1000	
1000–710	
710–500	
500–250	
250–125	
125–45	

### X-Ray Powder Diffraction (XRPD)

For X-ray powder diffraction measurements an X’Pert MRD Pro (PANalytical, Almelo, the Netherlands) was used in reflection mode at 45 mV and 40 mA with an X’Celerator detector and nickel filtered CuKα1 radiation. The scanning step size was 0.017° 2θ in a range of 5° to 45° 2θ.

### Differential Scanning Calorimetry (DSC)

DSC experiments were carried out on a Mettler-Toledo DSC 2 equipped with a nitrogen cooling system (Mettler-Toledo, Gießen, Germany). The glass transition temperatures of the ASDs were determined using a multi-frequency temperature modulation (TOPEM-mode) with an underlying heating rate of 2 K/min with a constant nitrogen purge (30 ml/min). Therefore, approximately 10 mg of the respective sample was accurately weighed into aluminum pans and sealed with a pierced lid.

### Fourier-Transform Infrared Spectroscopy (FT-IR)

Solid-state molecular interactions were assessed using a Bruker Alpha FT-IR spectrometer (Billerica, MA, USA) equipped with an attenuated total reflection (ATR) accessory. The spectral range was 400–4000 cm^−1^, and 24 scans were recorded for each sample. All raw materials, physical mixtures, and ASDs were measured.

### Phase Behavior Upon Immersion in Hydrochloric Acid

In order to investigate a possible phase separation of the L100–55-based formulations, the ASD powder was immersed in 0.1 M hydrochloric acid according to the acidic stage of the pH-shift dissolution experiment. Subsequently, the powder was dried under vacuum at 40 °C over night. Then, the dry ASD powder was assessed regarding solid state using DSC and FT-IR.

### Non-Sink Dissolution

Supersaturating non-sink dissolution studies were conducted using the MiniDissolution apparatus, a miniaturized USP II apparatus with a volume of 20 ml (28). The paddle speed was constant for all experiments at 100 rpm and the temperature was set to 37 °C. One dissolution experiment was conducted at a static pH of 6.8 using phosphate buffer for 3 h. Additionally, a pH-shift dissolution experiment was carried out starting at pH 1 using 0.1 M hydrochloric acid for 1 h, followed by an adjustment to pH 6.8 with 1.9 ml of a buffer concentrate consisting of 0.375 M phosphate buffer and 0.85 M sodium hydroxide. ASDs of each particle size fraction equivalent to 8 mg ketoconazole were used for the dissolution experiments (theoretical concentration of 400 μg/ml). The L100–55 ASD at a drug load of 10% was also filled into enteric capsules (Vcaps Enteric®) prior the pH-shift experiment.

The ketoconazole concentration was measured online using a diode array UV/VIS spectrophotometer (Agilent 8453, Agilent Technologies GmbH, Waldbronn, Germany) with correction for light scattering.

## Results and Discussion

### Solid State of the ASDs

**Fig. 1:**
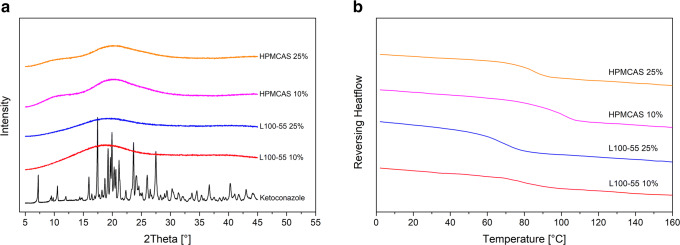
Solid state of the ASDs. X-ray powder diffraction (XRPD) diffractograms (**a**). Differential scanning calorimetry (DSC) thermograms (**b**)

The solid state was investigated using XRPD and DSC. For pure KTZ sharp reflection peaks within the diffractogram were observed due to its crystalline structure (Fig. 1a). The crystalline nature of KTZ was confirmed by DSC measurements which revealed a melting point at 147 °C. The ASDs comprising KTZ and either L100–55 or HPMCAS LG exhibited an amorphous halo within the diffractograms, indicating a complete transition of KTZ into the amorphous state. For all ASDs, single glass transition temperatures were observed. The glass transition temperatures of the L100–55-based ASDs at 10 and 25% drug load were found at 80.6 ± 1.2 °C and 73.0 ± 0.7 °C, respectively. The glass transition temperatures for the HPMCAS LG ASD at 10 and 25% drug load were found at 99.1 ± 0.6°C and 83.8 ± 0.6°C, respectively (Fig. 1b). The presence of single glass transition temperatures clearly indicated the formation of single-phased ASDs.

**Fig. 2:**
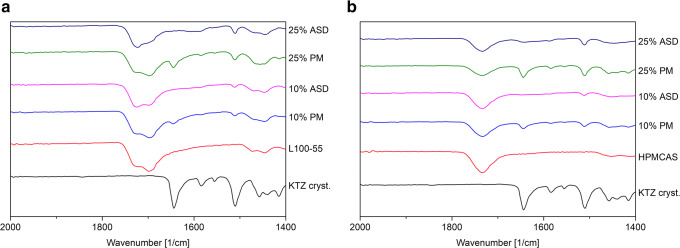
Fourier-transform infrared spectra of KTZ-L100–55 formulations (**a**) and KTZ-HPMCAS LG formulations (**b**). *PM* physical mixture

The FT-IR spectrum of pure KTZ showed characteristic bands at 2963 cm^−1^ and 2883–2831 cm^−1^ originated by CH-vibrations, a C=O stretching at 1644 cm^−1^ and an aromatic C=C stretching at 1509 cm^−1^. L100–55 exhibited a C=O double band at 1726 cm^−1^ (esterificated carboxylic groups) and 1698 cm^−1^ (carboxylic groups), whereby the latter band had a higher intensity. Pure HPMCAS LG showed a C=O stretching vibration at 1732 cm^−1^ (Fig. 2).

For the physical mixtures of L100–55 and KTZ, the characteristic C=O band from KTZ was still visible. This band completely vanished for the respective ASDs. Additionally, the shape of the C=O double peak from the L100–55 changed, whereby the peak at 1726 cm^−1^ intensified compared with the one at 1698 cm^−1^. These peak transitions clearly indicate specific interactions between KTZ and the polymer. For the HPMCAS-based ASDs ,similar observations could be made with regard to the peak of KTZ, which also vanished. However, no shift or shape change could be detected for the C=O band at 1732 cm^−1^, which also indicated formation of specific interactions, but without participation of the carbonyl moiety of HPMCAS LG (Fig. 2).

### pH-Dependent Dissolution Behavior of Ketoconazole

**Fig. 3:**
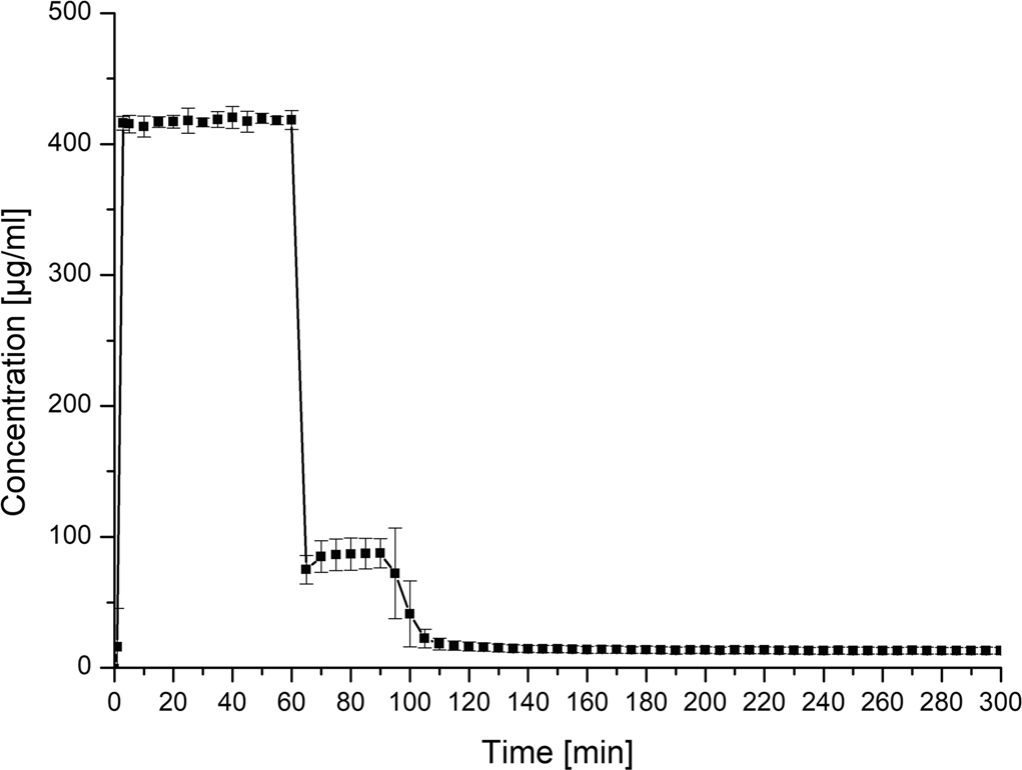
pH-shift dissolution experiment of neat KTZ (0–60 min: pH 1; 60–300 min: pH 6.8)

Ketoconazole is a weak base and consequently its dissolution behavior is expected to be highly pH-dependent. In order to investigate the pH-dependent dissolution behavior, a pH-shift experiment was conducted. The initial pH was 1, which was maintained for 1 hour to simulate the gastric stage; subsequently the pH was adjusted to pH 6.8 for 4 hours to mimic the intestinal stage. As apparent from Fig. 3, KTZ dissolved to full extent within the acidic stage due to the fact that KTZ was present in its ionized form as a result of its basic nature. Upon the pH adjustment to 6.8, a pronounced precipitation occurred to a concentration level of approximately 100 μg/ml. However, this concentration level represented a supersaturated state of KTZ relative to the saturation solubility, which was caused by the pH-shift. This state was maintained for approximately 40 min, which was followed by a further concentration drop to the level of the saturation solubility. Such a pH-dependent solubility profile can be considered as problematic when it comes to absorption of such a molecule because of the low solubility at the site of absorption. Consequently, it would be desirable to create a formulation that achieves higher concentration at pH 6.8.

### Dissolution Studies of the ASDs

In order to assess the dissolution performance of the ASDs, two types of dissolution experiments were performed. Initially, the ASDs were tested using a static pH of 6.8. This experimental setup was selected in order to investigate the performance of the ASDs at conditions that are present at the site of absorption with regard to the pH. The performance at these conditions can be considered as “best case scenario” since there is no further influencing factor that could alter/lower the performance of the ASDs. Consequently, the dissolution experiment at a static pH should provide insight of the theoretical performance of the ASDs at simulated site of absorption.

Subsequently, a pH-shift experiment was performed, which was supposed to be more representative for the physiological pH transition (from pH 1 to pH 6.8). The general aim was to minimize the release extent at acidic pH and maximize the release at pH of 6.8 (i.e., inverting of the dissolution behavior of pure KTZ). Therefore, pH-dependent soluble polymers were selected as ASD matrix, whose dissolution behavior is converse relative to pure KTZ. The potential to invert the dissolution behavior of KTZ was investigated as a function of polymer type, drug load, and particle size.

### Monophasic Non-Sink Dissolution at Static pH

**Fig. 4:**
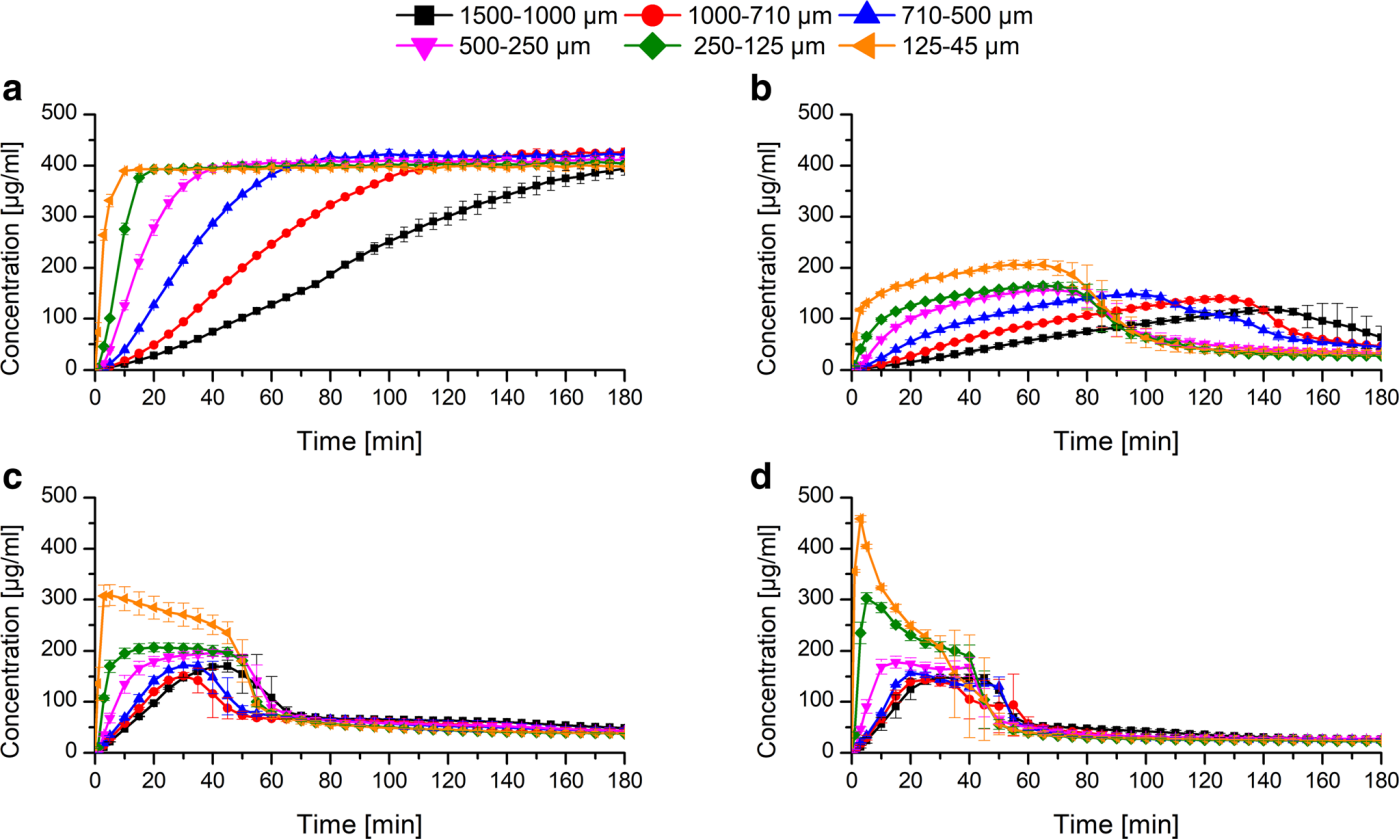
Non-sink dissolution experiment at pH 6.8 with different particle size fractions of ASDs of L100–55 (10% drug load) (**a**), L100–55 (25% drug load) (**b**), HPMCAS LG (10% drug load) (**c**), and HPMCAS LG (25% drug load) (**d**)

At first, the ASDs with six different particle fractions for each polymer and each drug load were tested in a dissolution test using a static pH of 6.8. Among the L100–55 ASD at a drug load of 10%, the dissolution rate was dependent on the particle size. With decreasing particle size, the dissolution rate increased due to a higher specific surface area according to the Noyes-Whitney equation (39). For every particle size fraction, the maximum concentration of 400 μg/ml was approached and maintained in solution without occurrence of precipitation. Consequently, the polymer L100–55 had excellent precipitation inhibitory properties upon dissolution of a 10% drug load ASD and provided a desirable dissolution profile for KTZ (Fig. 4a). Among the different particle sizes of the L100–55 ASD at 25% drug load, the rank order regarding the dissolution rate was dependent on the particle size analogous to the 10% drug load formulation. However, the maximum achieved concentration was only approximately 200 μg/ml for the finest fraction and 150 μg/ml for all other particle fractions. Additionally, precipitation occurred for all particle fractions, whereby a faster dissolution rate led to an earlier precipitation induction. This observation is in accordance with the study of Sun and Lee (2013) (32), as they found that the rate of supersaturation generation was proportional to the precipitation rate. The dissolution performance of the 25% drug load formulation was inferior compared with the 10% drug load as a consequence of a lower polymer concentration in solution upon dissolution of the ASD, which caused the inability to reach equally high concentrations and to maintain the supersaturated state (Fig. 4b). Several studies have shown that the polymer concentration is a critical factor for the level of supersaturation (i.e., amorphous solubility) and the course of the supersaturation over time. In most cases, both could be improved with an increased polymer concentration (i.e., lower drug load) (40–42).

For the HPMCAS-based ASDs, the initial dissolution behavior was also dependent on the particle size, whereby the dissolution rate increased with decreasing particle size as well. Among the 10% drug-loaded ASD particle fractions, the finest fraction resulted in the highest concentration level (~ 300 μg/ml). All other fractions yielded lower concentrations. After approximately 60 min of a plateau phase, the concentration dropped to ~ 50 μg/ml for all fractions (Fig. 4c). The HPMCAS-based ASDs with 25% drug load behaved very similar compared with the 10% drug load. Again, the dissolution rate was the fastest for the smallest fraction, and there was also a plateau phase with a subsequent drop in concentration to a level of 50 μg/ml after approximately 60 min (Fig. 4d). The similarity of the dissolution profiles revealed that the drug load (and total polymer concentration) had a minor impact on the dissolution rate and the supersaturation maintenance in the case of HPMCAS LG ASDs.

### pH-Shift Non-sink Dissolution

Following the dissolution experiments at the static pH (6.8), a pH-shift dissolution experiment was conducted. This experiment was based on two pH stages, initially at pH 1 for 60 min; subsequently the pH was adjusted to 6.8 which was maintained for 240 min. These conditions are supposed to mimic the pH transition in the human gastrointestinal tract. With this type of experimental setup, it was investigated to what extent the release of KTZ at acidic pH could be controlled via the particle size. Therefore, the least possible extent of release would be desirable, to avoid precipitation of KTZ upon pH-shift due to the absence of the polymer in solution as it is insoluble at acidic pH. Consequently, the best scenario would be that the release of KTZ at acidic pH is close to zero while providing a rapid and complete release at pH 6.8. Additionally, it was intended to provide insight into the influence of acidic exposure on the ASDs. In particular, the extent to which the acidic environment influenced the release at pH 6.8 was questioned.

**Fig. 5:**
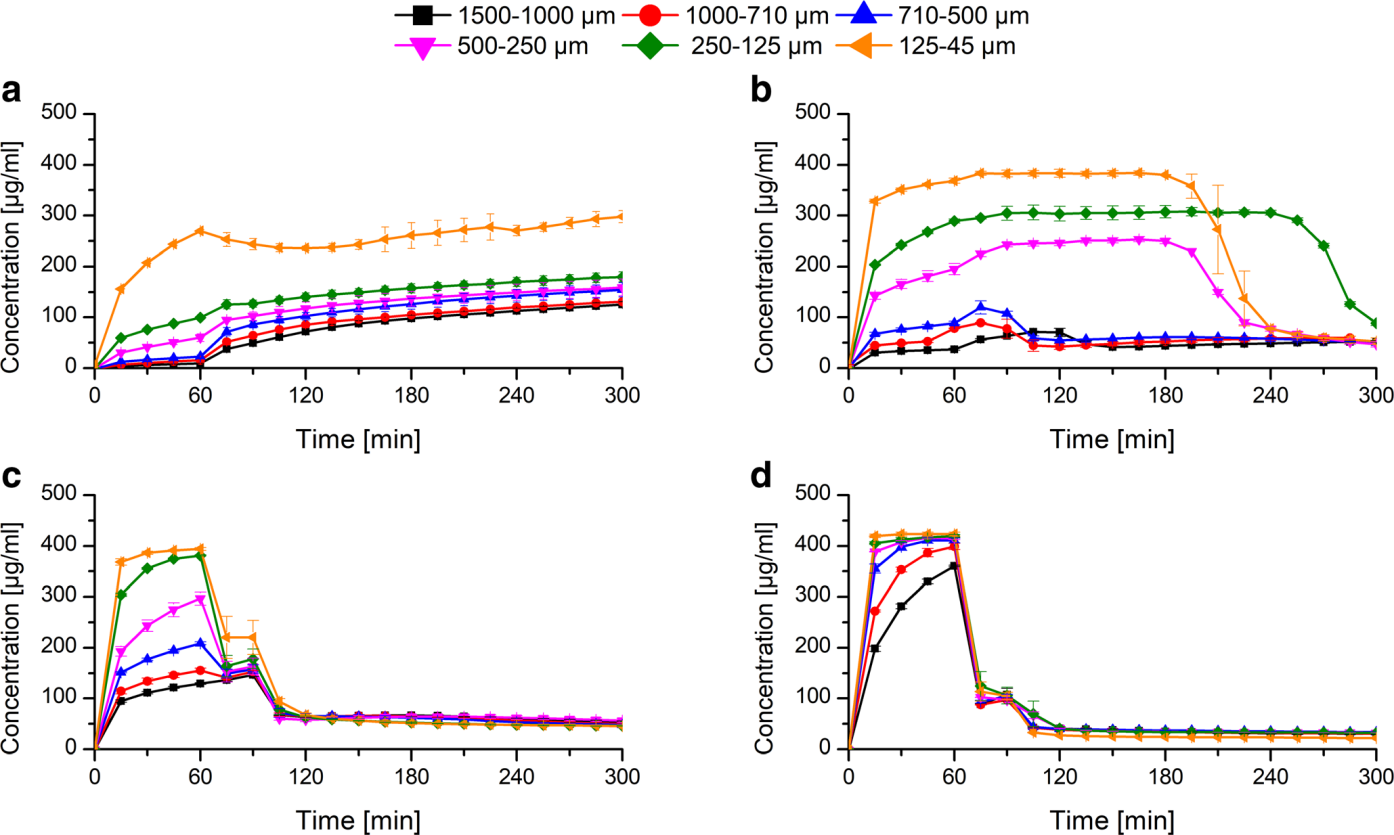
pH-shift non-sink dissolution experiment (0–60 min: pH 1; 60–300 min: pH 6.8) with different particle size fractions of ASDs of L100–55 at (10% drug load) (**a**), L100–55 (25% drug load) (**b**), HPMCAS LG (10% drug load) (**c**), and HPMCAS LG (25% drug load) (**d**)

As for the L100–55 ASDs at 10% drug load, almost no release of KTZ in the acidic stage was observed for the coarsest fraction (1500–1000 μm), while for the finest fraction (125–45 μm) a pronounced release was observed (9 μg/ml *vs.* 270 μg/ml) (Fig. 5a). In general, the release of KTZ at pH 1 was inversely proportional to the particle size. As the specific surface area of the ASD particles increased with decreasing particle size, more KTZ molecules were thus exposed to the acidic medium for the finer fractions compared with the coarser fractions. It has to be considered that the polymer is insoluble in acidic medium; consequently the release of KTZ was only originated by KTZ being present on the surface and by diffusion through the hydrated polymer.

Upon adjustment of the pH to 6.8, the polymer (L100–55) started to dissolve. An increase in dissolution rate could consequently be observed for all fractions, except for the finest fraction. However, the dissolution rate and the reached concentration plateau were markedly reduced, compared with the performance at the static pH (6.8), as it only reached approximately half of the concentration level. The finest fraction of the L100–55 (10% drug load) ASD deviated from the other particle fractions. After 60 min, the resulting concentration was already at 270 μg/ml and subsequent to the pH adjustment, and a slight drop in concentration could be observed, likely due to precipitation of KTZ as a result of its pH-dependent solubility (e.g., dissolution profile of neat KTZ). Additionally, immediately upon pH-shift, there was no polymer in solution, which could have stabilized the KTZ in solution. However, after 120 min, the concentration increased again to a final concentration of 297 μg/ml, possibly as a result of the dissolution of the remaining intact ASD or re-dissolution of KTZ in presence of the dissolving polymer. Conclusively, the coarse fractions of the 10% drug loaded L100–55 ASD effectively retained the release of KTZ within the acidic stage, but none of the particle fractions could achieve concentration levels as tested in the static dissolution experiment after the pH adjustment.

For the L100–55-based ASD at a drug load of 25%, the trend of the release of KTZ at the acidic stage was similar to the 10% drug load ASD (Fig. 5b). The extent was higher for each respective particle fraction since the higher drug load led to the fact that relatively more KTZ molecules were present on the ASD surface. However, for the three fine particle fractions (125–45 μm; 250–125 μm; 500–250 μm), no concentration decrease occurred after the pH adjustment despite the fact that at this time point, only very a small amount of polymer (L100–55) was dissolved. Due to the high surface area of the finer particles, the polymer could dissolve very rapidly, which in turn could stabilize the supersaturated KTZ. As revealed in the static dissolution experiment, the polymer concentration was not sufficient to maintain the supersaturated level for the whole period; as a result precipitation occurred within the pH-shift experiment, too. For the three coarser fractions, there was a limited release at pH 1 and also upon pH-shift, the concentration level remained on a low level, and no increase in dissolution rate was observed (Fig. 5b).

While the coarse particle fractions of the HPMCAS-based ASDs at 10% drug load effectively retained the release of KTZ (Fig. 5c), at a drug load of 25%, more KTZ was released at acidic pH due to relatively more KTZ molecules on the particle surface (Fig. 5d). With decreasing particle size, the dissolution profile for both drug loads approached the one of pure KTZ since the influence of the pH-dependent soluble polymer decreased with increasing surface area. After the pH-shift all HPMCAS-based ASD particle fractions showed a quantitative precipitation to the level of the solubility of KTZ as already observed by the static dissolution experiment. Consequently, the application of HPMCAS-based ASDs had no beneficial effect on the dissolution profile compared to pure KTZ because HPMCAS LG did not exhibit sufficient precipitation inhibiting effects. In order to potentially enhance the dissolution performance of HPMCAS-based ASDs, suitable surfactants might be added. It was shown that surfactants such as sodium lauryl sulfate or different types of poloxamer were able to improve the dissolution properties of HPMCAS-based ASDs (43,44).

### Phase Separation upon Immersion in Hydrochloric Acid

**Fig. 6:**
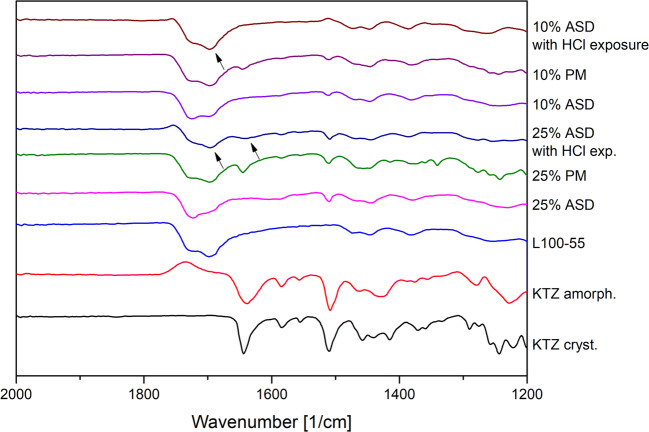
Fourier-transform infrared spectra of crystalline KTZ, amorphous KTZ, neat L100–55, physical mixtures and L100–55 ASDs (after preparation and after treatment with HCl)

Due to the fact that the dissolution rate of the L100–55 ASDs was markedly reduced at pH 6.8 when they were exposed to acidic medium, the phase behavior was investigated. Therefore, the ASD particles of both drug loads were immersed in 0.1 M hydrochloric acid for 60 min according to the pH-shift dissolution experiment. Subsequently, the particles were dried and analyzed using FT-IR and DSC. Figure 6 shows the FT-IR spectra of the ASDs, the respective physical mixtures, and the HCl-treated ASDs. Within the FT-IR spectrum of the HCl-treated ASD (25% drug load), the C=O peak at 1644 cm^−1^ re-appeared, which was not present within the spectrum of the ASD but in the spectrum of the physical mixture. Additionally, the peak shape of the C=O double peak at 1698 cm^−1^ and 1732 cm^−1^ re-shaped towards the pattern of the physical mixture (indicated by the arrows in Fig. 6). The re-shaping of the C=O double peak was also detected for the 10% drug-loaded ASD. These changes within the FT-IR spectra are indicative for an amorphous-amorphous phase separation (AAPS) within the ASD and a disruption of interactions between drug and polymer. Thereby, a drug-rich phase and a polymer-rich phase were formed (45).

**Fig. 7:**
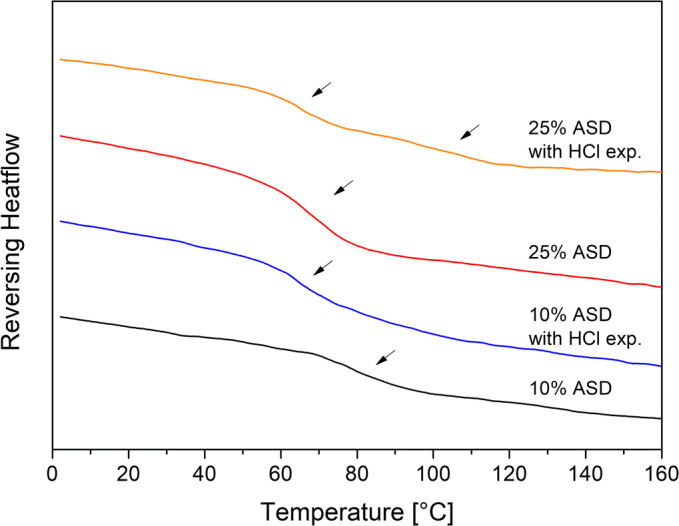
Differential scanning calorimetry (DSC) thermograms of the L100–55 ASDs at 10 and 25% drug load after preparation and after treatment with HCl. Glass transition temperatures are indicated by the arrows

The DSC measurement revealed changes in the glass transitions temperatures of the HCl-treated ASDs. Within the thermogram of the 25% drug loaded ASD, two distinct glass transition temperatures at 62.3°C and 101.4°C were found, which differed from the glass transition temperature of the untreated ASD (73.0°C). For the 10% drug load ASD, a shift of the glass transition temperature from 80.6°C (untreated) to 68.3°C was found (Fig. 7). The shift of the glass transition temperatures towards lower temperatures and the occurrence of two distinct glass transition temperatures also indicated a formation of an AAPS and a disruption of interactions between drug and polymer. The lower glass transition temperature reflected the drug-rich phase and the higher one corresponded to the polymer-rich phase (46,47).

The phenomenon of AAPS has been shown to be facilitated in presence of moisture as a consequence of an increased molecular mobility of the drug within the polymeric matrix (48,49). The formation of a drug-rich phase and a polymer-rich phase has been shown to negatively influence the dissolution behavior as upon AAPS, a congruent release of drug and polymer could turn into an undesirable incongruent release of drug and polymer (50,51). An incongruent release could lead to the fact that the dissolution is drug-controlled which was shown to profoundly decrease the dissolution rate of an ASD (48,52). These findings are in line with the observations within our study and explain the decreased dissolution rate after exposure to acidic medium. Despite the fact, that the release of KTZ at acidic pH could be effectively controlled by the particle size, the dissolution rate after pH shift was markedly reduced as a result of the occurrence of AAPS.

### pH-Shift Non-sink Dissolution Using Enteric Capsules

By solely adjusting the particle sizes of the L100–55 ASDs, it was not possible to achieve an optimal dissolution behavior (i.e., close to zero release at acidic pH and rapid and complete release at neutral pH), due to moisture-induced formation of a drug-rich and polymer-rich phase within the acidic stage. In order to avoid the exposure of the ASD particles to the acidic medium within the first dissolution stage, the 10% drug load L100–55 ASDs were filled into Vcaps Enteric®. As these capsules consist of the polymer HPMCAS LG, they are supposed to be insoluble in acidic medium and would therefore be capable to protect the ASD particles from the acidic medium.

**Fig. 8:**
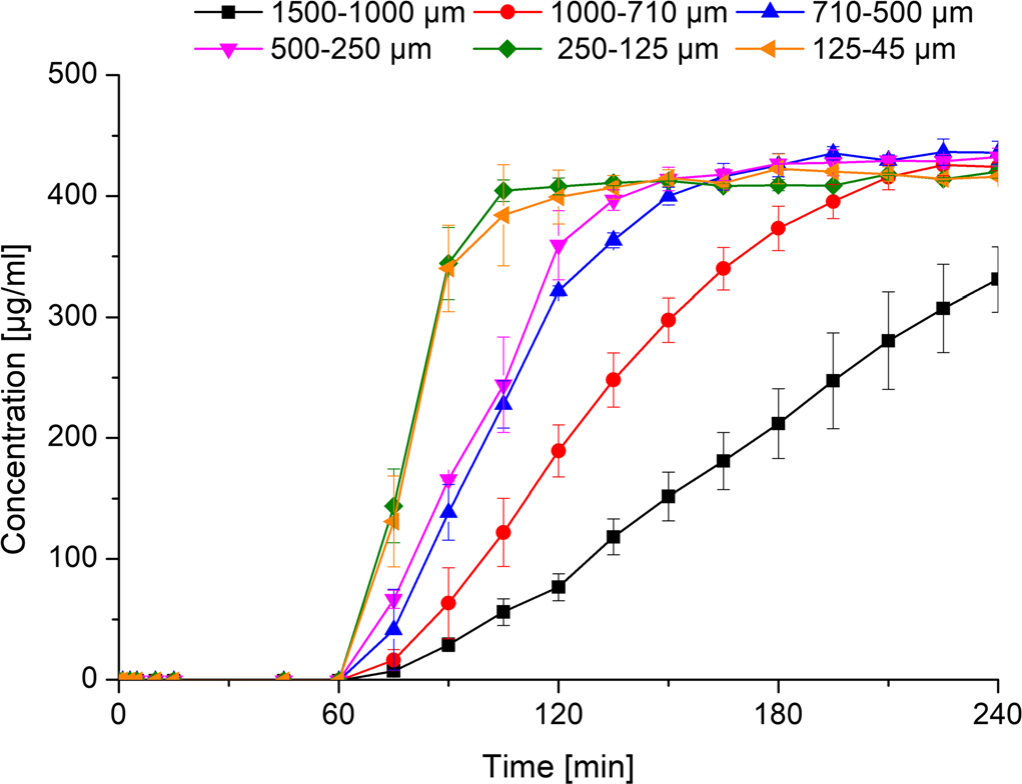
pH-shift non-sink dissolution experiment (0–60 min: pH 1; 60–240 min: pH 6.8) with different particle size fractions of ASDs of L100–55 at (10% drug load) filled into Vcaps Enteric®

Figure 8 shows the dissolution results of the enteric capsules using the pH-shift method. Within the acidic stage, no realease of KTZ took place. Following the pH-shift to pH 6.8, the capsule started to dissolve and the ASD particles were released. The dissolution behavior of the different ASD particle fractions resembled the dissolution profiles obtained in the static pH dissolution experiment, showing a complete and rapid release depending on the particle sizes and additionally an absence of precipitation. This suggests that the enteric capsules effectively protected the ASD from the acidic medium and as a consequence the undesirable phase separation could be avoided, which led to dissolution properties as desired at elevated pH. These findings highlight the potential of the application of enteric capsules for ASDs in general to ensure a sufficient dissolution performance by protecting the ASD formulations from adverse influencing factors such as acidic exposure.

## Conclusion

In this study, we successfully prepared ASDs at two drug loads (10% and 25%) using Eudragit L100–55 and HPMCAS LG as pH-dependent soluble polymers and KTZ as weakly basic model drug by hot-melt extrusion. KTZ was transformed into its amorphous state and single-phase ASDs with specific interactions between drug and polymer were formed, as indicated by XRPD, DSC, and FT-IR measurements. The ASDs were then milled to obtain six different particle fractions. Subsequently, the influence of particle size, drug load and polymer type on the dissolution performance was studied. Initially, the dissolution performance was studied at a static pH of 6.8, which revealed the most promising results for the L100–55 ASD at a drug load of 10% as a complete dissolution and a stable supersaturation without precipitation could be obtained. Thereby, the dissolution rate was inversely proportional the particle size. The dissolution of the L100–55 ASD at a drug load of 25% was markedly lower and the supersaturated level could not be maintained as a consequence of a lower polymer concentration in solution. Among the HPMCAS-based ASDs, a rapid initial release could be observed, but quantitative precipitation occurred in the course of the experiment, indicating a low precipitation inhibitory potential. In the following, a pH-shift dissolution experiment (from pH 1 to pH 6.8) was conducted to assess the influence of the acidic medium on the ASDs in general and specifically if the release of the weak base KTZ within the acidic stage could be controlled by the particle size. It was found that the most effective retaining of the release of KTZ in the acidic medium could be achieved by coarser particle fractions of the L100–55-based ASDs (close to zero for the coarsest particle fraction) as compared to the HPMCAS-based ASDs. However, following the pH-shift to pH 6.8, the dissolution rate of the L100–55 ASD at 10% drug load was markedly reduced compared to the static dissolution experiment for all particle fractions. This was due to the fact that an AAPS into a drug-rich phase and polymer-rich phase occurred during the exposure of ASDs to the acidic medium, which was identified by FT-IR and DSC. The occurrence of AAPS could successfully be prevented using enteric capsules (Vcaps Enteric®) since the ASDs were protected from the acidic medium. As a result, an optimal dissolution performance could be achieved for the L100–55-based ASD at a drug load of 10%.
